# Combined macular buckle and intraoperative OCT-guided 41-gauge drainage of refractory large bacillary layer detachments in neovascular age-related macular degeneration and diabetic macular edema

**DOI:** 10.1186/s40942-026-00875-6

**Published:** 2026-06-10

**Authors:** Ginevra Giovanna Adamo, Antonio Cartabellotta, Pietro Maria Talli, Giuseppe Giannaccare, Chiara Vivarelli, Marco Pellegrini, Francesco Parmeggiani, Marco Mura

**Affiliations:** 1 Department of Translational Medicine, University of Ferrara, Ferrara, Italy; 2 Sant’Anna University Hospital, Ferrara, Italy; 3 Eye Clinic, Department of Surgical Sciences, University of Cagliari, Cagliari, Italy; 4 King Khaled Eye Specialist Hospital, Riyadh, Saudi Arabia

**Keywords:** Subretinal drainage, Macular buckle, Bacillary layer detachment, Neovascular age-related macular degeneration, Diabetic macular edema, Intraoperative-OCT

## Abstract

**Background:**

To describe a novel combined surgical technique for the drainage of large bacillary layer detachment (BLD) in patients with advanced neovascular age-related macular degeneration (nAMD) or diabetic macular edema (DME) refractory to intravitreal anti-vascular endothelial growth factor (anti-VEGF) or corticosteroid therapy.

**Methods:**

This prospective pilot case series evaluated three eyes of three patients (two nAMD, one DME) with large BLDs runresponsive to intravitreal treatment. The standardized procedure consisted of ab-externo macular buckle placement followed by 23-gauge pars plana vitrectomy (PPV). Intraoperative OCT (iOCT) guided subretinal fluid drainage using a 41-gauge cannula. Primary outcomes were anatomical BLD resolution and changes in best-corrected visual acuity (BCVA) at six-month.

**Results:**

Immediate and complete anatomical resolution was achieved in all eyes. BCVA modestly improved from 20/400, 20/400, and 20/800 at baseline to 20/200, 20/200 and 20/400 Snellen at six-month follow-up. No intra- or postoperative complications occurred, and no additional anti-VEGF injections were required during follow-up.

**Conclusions:**

Combined intraoperative OCT-guided subretinal drainage with macular buckling achieved immediate anatomical resolution of large, chronic bacillary layer detachments refractory to medical therapy. Functional recovery was limited, likely reflecting irreversible outer retinal and retinal pigment epithelium damage in advanced disease. This approach may represent a salvage option in carefully selected cases of refractory exudative macular disease. Further studies with larger cohorts and longer follow-up are required to define its long-term efficacy, optimal timing, and clinical role.

**Supplementary Information:**

The online version contains supplementary material available at 10.1186/s40942-026-00875-6.

## Background

Bacillary layer detachment (BLD) has emerged as a distinctive tomographic entity, characterized by a longitudinal splitting within the photoreceptor inner segments. Specifically, this cleavage occurs at the level of the myoid zone, immediately posterior to the external limiting membrane (ELM). High-resolution optical coherence tomography (OCT) demonstrates that the internal boundary of these cystoid cavities is formed by the ELM and myoid zone, while the external boundary consists of the ellipsoid zone (EZ) and photoreceptor outer segments, which typically remain adherent to the underlying retinal pigment epithelium (RPE). Although BLD is not pathognomonic for any disease, it has been primarily identified across a broad spectrum of inflammatory and exudative disorders affecting the retina, including retinochoroiditis due to Toxoplasma, Vogt-Koyanagi-Harada disease, and severe ocular trauma [1–3] and in advanced neovascular age-related macular degeneration (nAMD) [4–7] and diabetic macular edema (DME) [8, 9]. 

In the context of nAMD, BLD is often a hallmark of severe exudative activity. Reported incidence rates of BLD in nAMD range from 4.5% to 7.4%, with a higher prevalence observed in eyes harboring mixed Type 1 and Type 2 macular neovascularization, extensive fluid accumulation, EZ attenuation, and sub-RPE involvement [4, 7, 10]. 

Despite the efficacy of intravitreal anti-vascular endothelial growth factor (anti-VEGF) therapy—the current gold standard—a subset of patients develops refractory large-volume BLD. In these cases, the accumulation of dense, often fibrinous subretinal material and fluid creates a mechanical barrier that persists despite aggressive “treat-and-extend” regimens [11]. Chronic separation of the photoreceptor cell bodies from the EZ leads to progressive metabolic stress and irreversible atrophy, necessitating a shift from purely pharmacologic to mechanical therapeutic strategies.

Similarly, in DME, BLD represents an extreme manifestation of outer retinal disruption, particularly in cases refractory to conventional medical management. Chronic vascular leakage and Müller cell dysfunction, exacerbated by outer retinal ischemia, are thought to contribute to the development of BLD. Notably, BLD in DME has been reported in patients unresponsive to standard-of-care intravitreal injections (both anti-VEGF and corticosteroids), suggesting that BLD may represent a marker of advanced or treatment-resistant disease with limited responsiveness to pharmacologic interventions alone [8, 9]. 

Currently, the management of large, persistent BLD remains a significant clinical challenge. There are no standardized or validated protocols for cases where medical therapy fails to achieve anatomical flattening. As the duration of detachment and ongoing exudation is directly correlated with progressive and irreversible photoreceptor damage and “foveal burnout,” a more proactive approach may be warranted. In this case series, we describe a novel multimodal surgical strategy to address the mechanical sequestration of fluid in refractory BLD. Our approach combines the ab-externo support of a macular buckle with the ab-interno precision of intraoperative OCT (iOCT)-guided 41-gauge drainage of large cystoid BLDs. By integrating these techniques, we aim to provide immediate anatomical restoration and prevent the long-term structural collapse of the macular architecture in advanced nAMD and DME.

## Methods

**Fig. 1:**
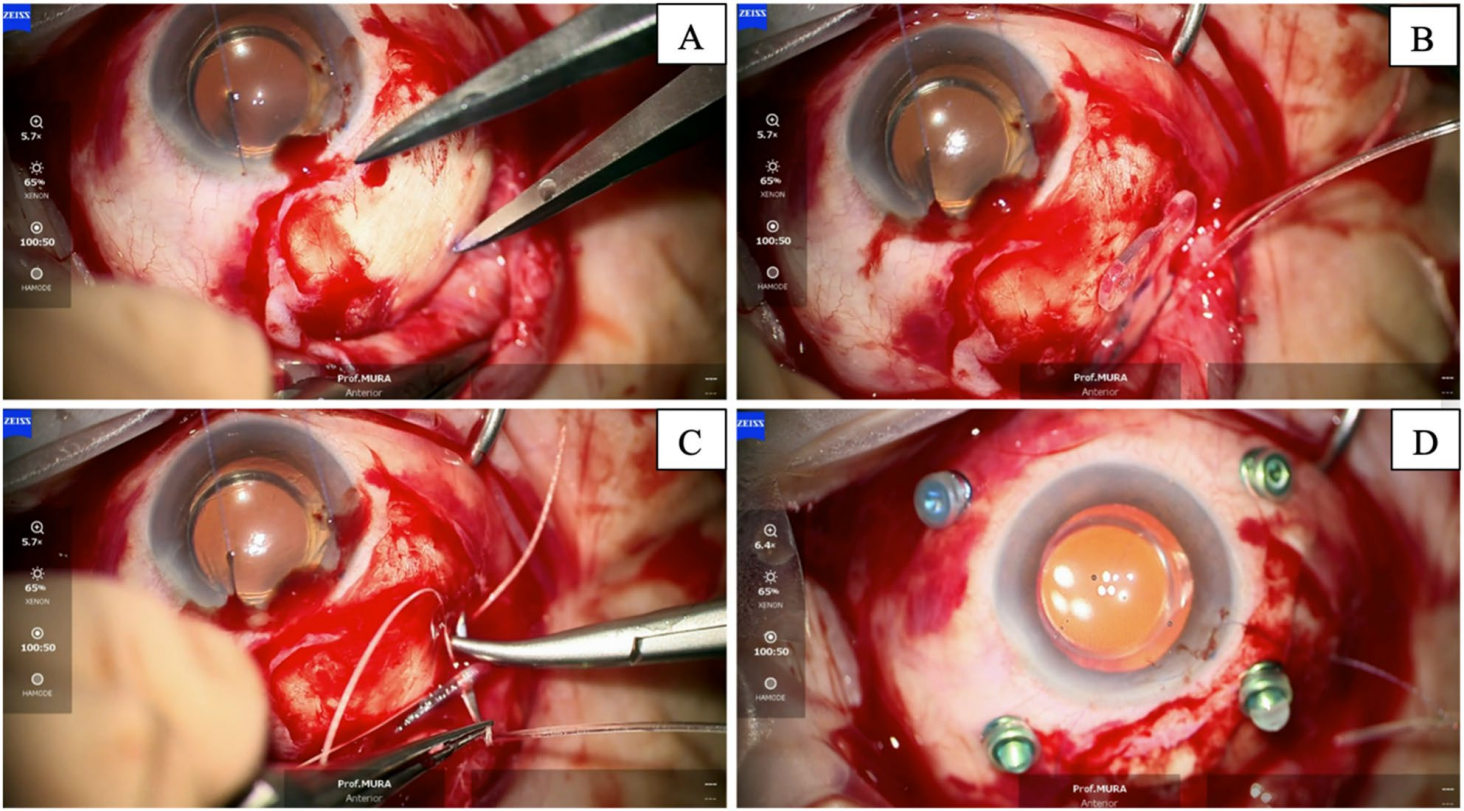
Placement of the customized scleral buckle at 12 mm from the limbus (**A**) to (**C**). Positioning of 23-gauge trocars for PPV (**D**)

This prospective interventional case series describes a novel surgical technique for the drainage of large cystoid refractory BLDs in patients with advanced nAMD and DME. Refractory BLD was defined as persistence of a large bacillary layer detachment despite previous intravitreal treatments (anti-VEGF and/or corticosteroids) without meaningful anatomical improvement on OCT. All surgical procedures were performed at Sant’Anna University Hospital, University of Ferrara, Italy. The study was conducted in accordance with the tenets of the Declaration of Helsinki, and written informed consent was obtained from all patients after a detailed explanation of the off-label nature of the procedure and its potential risks and benefits. Two patients with end-stage nAMD and one patient with advanced DME were included. Surgical candidacy was limited to eyes demonstrating: a large, well-demarcated cystoid BLD on OCT, persistent and clinically significant central visual disturbance, and absence of response to intravitreal injections. Eyes with diffuse or poorly defined intraretinal fluid were not considered suitable candidates. Exclusion criteria included also active ocular inflammation and uncontrolled glaucoma. Baseline assessments comprised best-corrected visual acuity (BCVA), slit-lamp biomicroscopy, dilated fundus examination, intraocular pressure (IOP) measurement, and macular OCT imaging. All procedures were performed by the same experienced vitreo-retinal surgeon. A surgical video is provided as Supplemental Digital Content 1, which highlights the key steps of the the surgical procedure. The ab externo technique involved the placement of a customized macular buckle (Fig. 1).

**Fig. 2:**
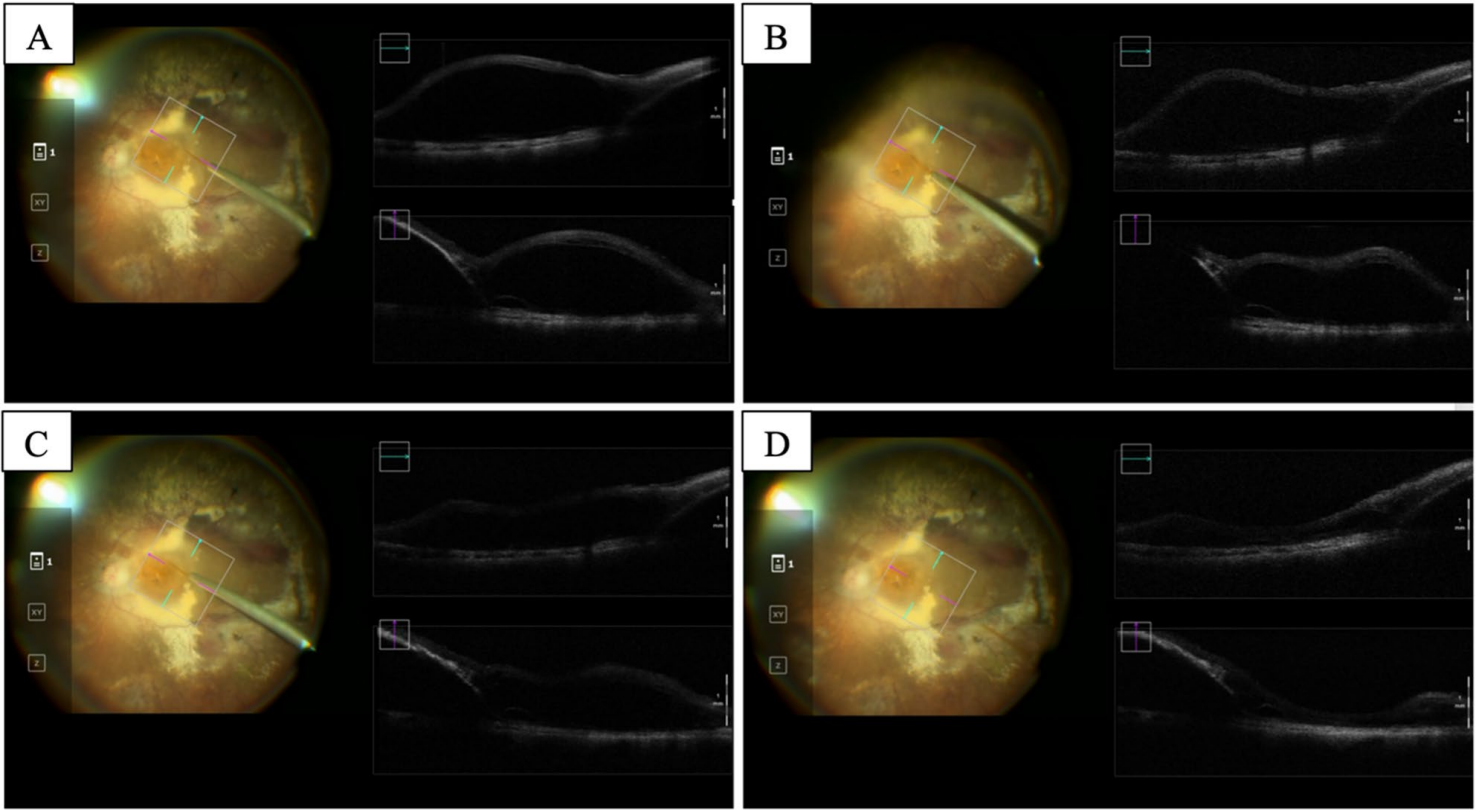
Intraoperative OCT assisted drainage through the 41-gauge needle. From (**A**) to (**D**) progressive deflation of the fluid filled cyst

A superior temporal conjunctival peritomy was created, followed by careful dissection of Tenon’s capsule from the sclera. Diathermy was applied to achieve adequate scleral exposure. The buckle was positioned in the superotemporal quadrant, with its head placed beneath the macula. The anterior arm of the buckle was sutured to the sclera 12 mm posterior to the limbus using 5 − 0 polyester sutures. Correct intraoperative positioning of the buckle was confirmed using a single-fiber chandelier light and visualization through the surgical microscope. Subsequently, a chandelier-assisted 23-gauge pars plana vitrectomy was performed. Following core and peripheral vitrectomy, a 41-gauge subretinal cannula was used to puncture the large cystoid serous space and drain the intraretinal fluid. Intraoperative OCT (Carl Zeiss Meditec AG. Artevo 800 Surgical Microscope. Oberkochen, Germany) provided real-time guidance during the drainage, confirming a complete flattening of the BLD. Throughout the subretinal maneuver, the retina was directly visualized, and iOCT images were continuously acquired (Fig. 2).

A fluid-air exchange was then performed, and the sclerotomies were closed with 7 − 0 Vicryl sutures. Postoperative treatment consisted of topical antibiotics and corticosteroids tapered over four weeks. Patients were examined on postoperative days 1 and 7, and at 1, 3 and 6 months. Follow-up evaluations included distance BCVA, IOP measurement, slit lamp and fundus examination, and macular OCT imaging to assess BLD resolution.

## Results

Three patients with bilaterally advanced retinal disease refractory to anti-VEGF therapy were included: a 76-year-old female (Patient 1) and two males aged 69 and 82 years (Patients 2 and 3). Table 1 shows the characteristics of the three patients.

**Table 1:** Characteristics of patients who underwent combined macular buckle and OCT-guided drainage for refractory bacillary layer detachments

Parameter	Patient 1	Patient 2	Patient 3
Age (years)	76	69	82
Gender	Female	Male	Male
Lens status	Pseudophakic	Pseudophakic	Phakic
Axial Length (mm)	23.18	22.75	23.98
Baseline BCVA (Snellen)	20/400	20/400	20/800
6-months post-op BCVA (Snellen)	20/200	20/200	20/400
Follow-up (months)	6	6	6
IOP (mmHg)	16	18	14
Macular Pathology	nAMD	DME	nAMD

BCVA: best-corrected visual acuity. nAMD: neovascular age-related macular degeneration. DME: diabetic macular edema. IOP: intraocular pressure

### Patient 1

**Fig. 3:**
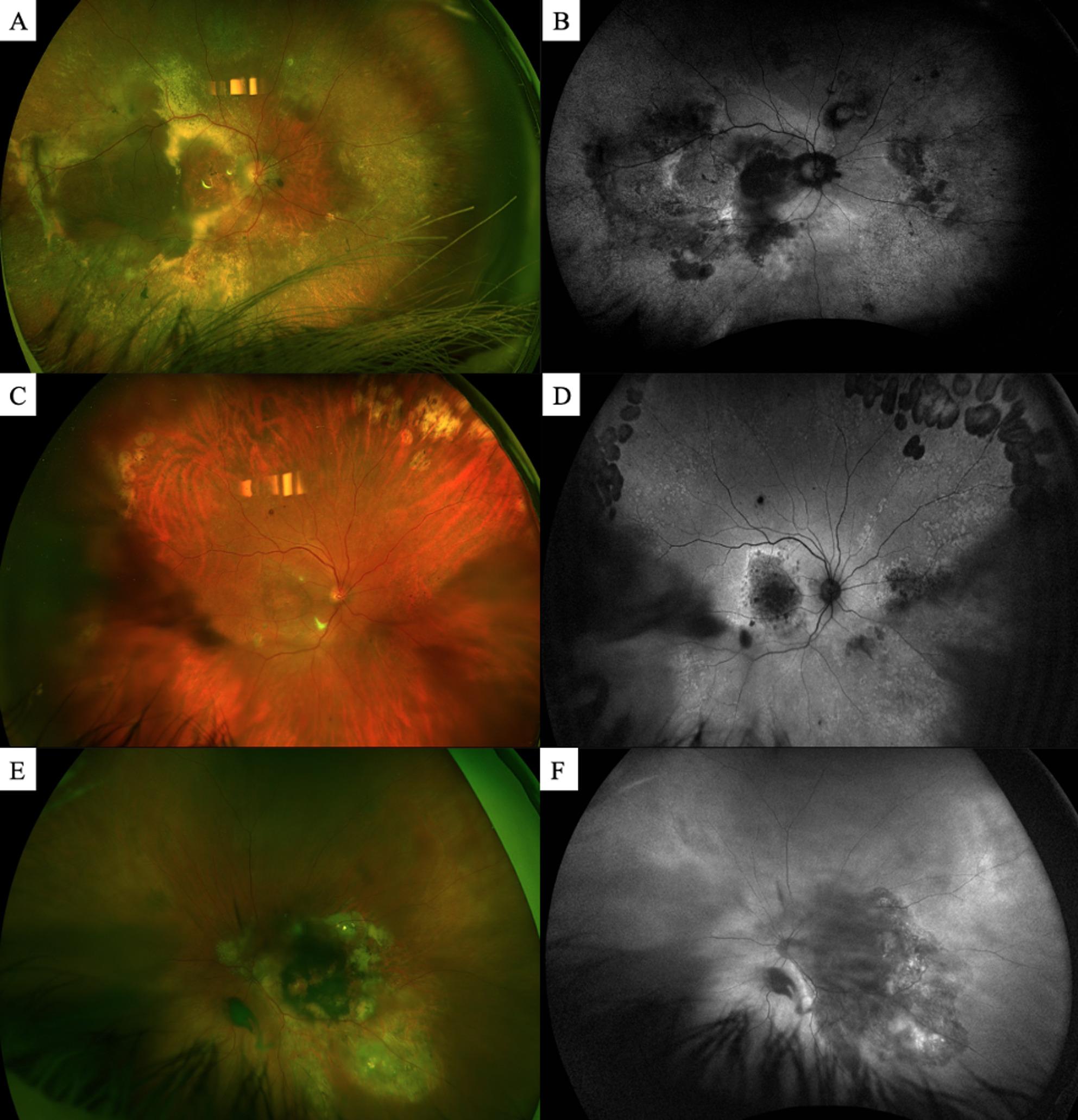
Pre-operative color fundus picture and fundus autofluorescence of patient 1 (**A**, **B**); patient 2 (**C**, **D**); and patient 3 (**E**, **F**)

**Fig. 4:**
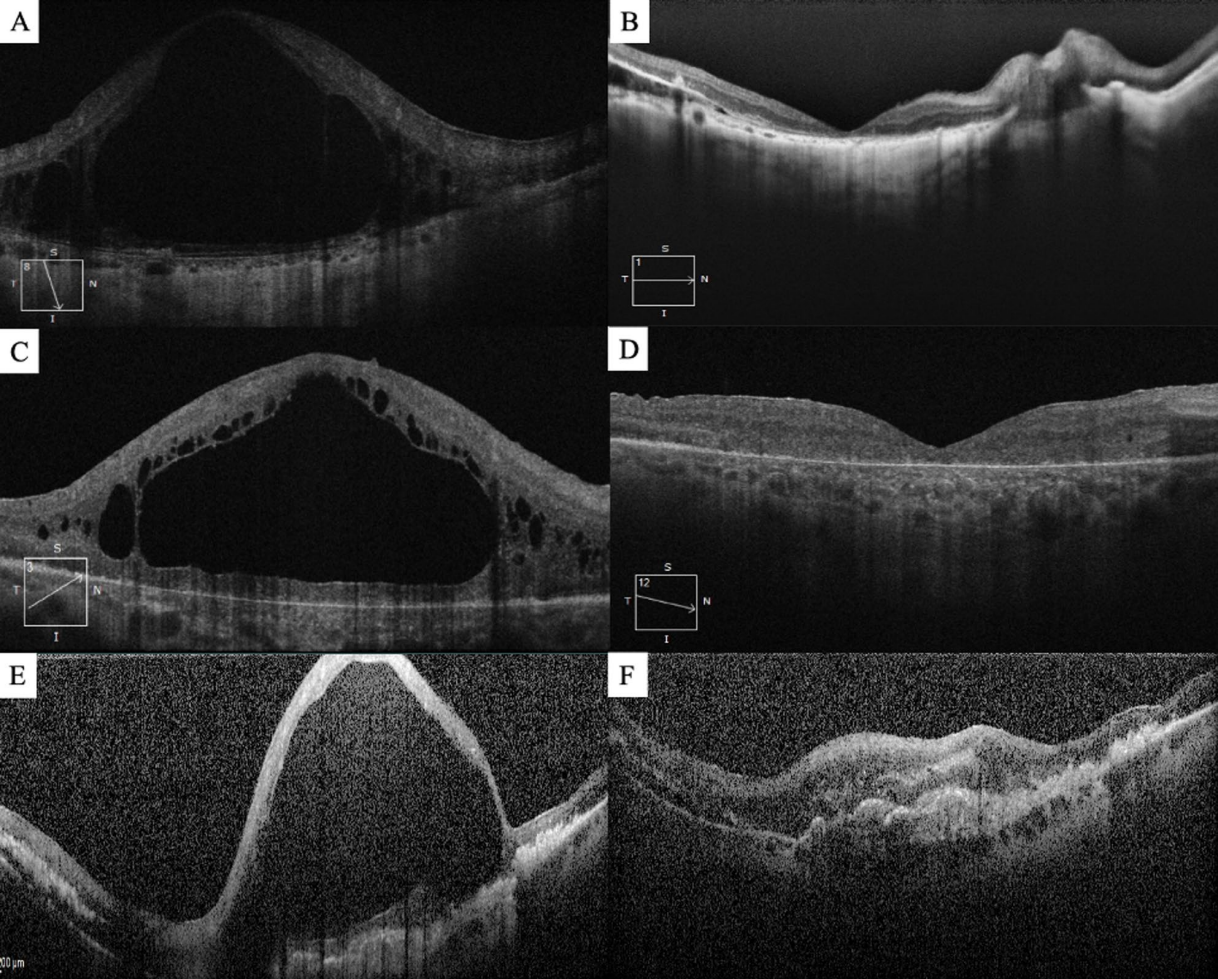
Pre-operative and 6-months after surgery OCT B-scans of patient 1 (**A**, **B**); patient 2 (**C**, **D**); and patient 3 (**E**, **F**)

A 76-year-old Caucasian female with bilateral nAMD. At presentation, best-corrected visual acuity (BCVA) was 20/400 Snellen in the right eye and < 20/800 Snellen in the left eye. Both eyes had previously received multiple intravitreal anti-VEGF injections. Color fundus imaging (Silverstone, Optos, CA, USA) of the right eye revealed macular schisis and extensive exudation (Fig. 3A), while spectral-domain optical coherence tomography (SD-OCT) demonstrated a large serous cystoid BLD adjacent to a serous pigment epithelial detachment (PED) and severe disruption of the outer retinal layers (Fig. 4A). The contralateral eye exhibited a disciform scar.

### Patient 2

A 69-year-old Caucasian male with bilateral DME presented with progressive vision loss in the right eye. BCVA was 20/400 in the right eye and 20/50 in the left eye. The left eye remained controlled with anti-VEGF therapy every eight weeks, whereas the right eye was refractory to multiple prior anti-VEGF and corticosteroid injections. Fundus examination and color fundus imaging of the right eye revealed macular schisis and marked exudation (Fig. 3C), and SD-OCT showed a serous BLD associated with multiple intraretinal cysts and pronounced outer retinal disruption (Fig. 4C).

### Patient 3

An 82-year-old Caucasian male with bilateral nAMD. BCVA at presentation was 20/800 in the left eye and < 20/800 in the right eye. Both eyes had previously received multiple intravitreal anti-VEGF injections. Fundus imaging of the left eye demonstrated advanced nAMD with fibrosis and atrophy (Fig. 3E), and SD-OCT revealed a large cystoid BLD overlying a fibrotic scar, with associated perilesional exudation and severe outer retinal compromise (Fig. 4E). The contralateral eye exhibited a macular hole secondary to pre-existing nAMD.

At six months postoperatively, SD-OCT B-scans confirmed resolution of subretinal and intraretinal fluid in all treated eyes (Fig. 4B, D, F). Fundus examination showed retinal reattachment, and intraocular pressure remained within normal limits. BCVA improved in eccentric fixation from 20/400, 20/400, and 20/800 at baseline to 20/200, 20/200, and 20/400 Snellen in Patients 1,2, and 3, respectively. All patients reported subjective improvement in central visual disturbances, including perceived reduction in central scotoma, and described an improvement in their daily life activities following surgery; however, no objective functional assessments (such as microperimetry or visual field testing) were performed to corroborate these findings.

## Discussion

BLD represents a distinctive OCT finding characterized by a schisis within the photoreceptor inner segment myoid zone, resulting in elevation of the ELM while the EZ and photoreceptor outer segments remain adherent to the RPE. This specific splitting pattern reflects an intrinsic structural vulnerability of the inner segment myoid region and facilitates the accumulation of intraretinal fluid in a cystoid configuration, thereby distinguishing BLD from other forms of macular edema. Although BLD has been described across a wide spectrum of inflammatory and exudative retinal disorders - such as Vogt–Koyanagi–Harada disease, central serous chorioretinopathy, and nAMD - its presence consistently reflects substantial extracellular fluid shifts and severe disruption of outer retinal architecture [12]. 

In nAMD, BLD has been identified in a clinically relevant subset of eyes undergoing detailed imaging evaluation. Data from a phase III clinical trial demonstrated that classic BLD occurred in approximately 7.2% of eyes with nAMD and was associated with worse baseline visual acuity, increased central subfield thickness, greater volumes of subretinal fluid and subretinal hyperreflective material (SHRM), and more extensive EZ attenuation compared with eyes without BLD. Although anti‑VEGF therapy resulted in anatomical resolution of BLD in nearly 98% of cases, visual acuity improvements remained inferior compared with non-BLD eyes, suggesting that resolution of fluid does not fully reverse the underlying outer retinal damage once structural integrity has been compromised [5]. 

A similar pattern has been observed in DME, where BLD is typically associated with severe exudation and substantial foveal thickening. In treatment‑naïve DME, BLD has been reported in approximately 6.2% of eyes and was associated with significantly increased baseline central foveal thickness. While anti‑VEGF therapy often resulted in apparent anatomical resolution of BLD, subsequent OCT imaging frequently demonstrated persistent disruption of the EZ and interdigitation zone, indicating that chronic intraretinal fluid accumulation may result in lasting outer retinal damage despite flattening of the macula [8]. Isolated case reports describing BLD in diabetic retinopathy treated with anti-VEGF have further implicated underlying choroidal ischemia and vascular compromise in its pathogenesis [9]. 

The management of BLD remains particularly challenging in chronic cases refractory to standard intravitreal therapy. In advanced nAMD and severe DME, persistent exudation may continue despite repeated anti‑VEGF or corticosteroid injections, leading to the development of large, stable cystoid cavities within the bacillary layer. In this scenario, no established alternative treatment strategy is currently available. In selected refractory cases, surgical drainage has been proposed as a potential intervention. Yiu et al. reported pars plana vitrectomy with subretinal cannula aspiration under iOCT guidance in Coats disease, achieving immediate anatomical collapse of the cystoid spaces; however, functional recovery was limited by disease chronicity [13]. Likewise, Ishikawa et al. described intraretinal cyst fluid aspiration in anti-VEGF-resistant DME, demonstrating anatomical improvement with partial visual recovery [14]. Collectively, these reports suggest that direct surgical drainage of fluid may be feasible in selected refractory cases, particularly when large cystoid spaces persist despite optimized medical therapy. To our knowledge, this is the first report describing a combined ab interno drainage and ab externo macular support approach targeting large, treatment refractory BLD in exudative macular disease. Importantly, the primary objective of this intervention is the mechanical resolution of persistent cystoid cavities and the potential alleviation of central visual disturbances in carefully selected symptomatic patients. In the present case series, our strategy integrates iOCT–guided subretinal drainage using a 41-gauge cannula with adjunctive macular buckling. The iOCT enables real-time confirmation of precise and controlled evacuation of the cystoid cavity, with immediate anatomical collapse. The addition of a macular buckle is intended to flatten the posterior sclera, thereby modifying its curvature and providing biomechanical support of the macular region. This maneuver reduces mechanical stress on the outer retina and may limit recurrence of fluid accumulation or further splitting of the photoreceptor IS. Previous studies have consistently demonstrated that macular buckling effectively alters macular biomechanics and promotes retinal anatomical reattachment in complex tractional conditions. In a randomized clinical trial, Liu et al. [15] showed that macular buckling achieved superior anatomical outcomes and lower surgical failure rates compared with pars plana vitrectomy alone in highly myopic eyes with macular schisis and associated macular detachment, underscoring the critical role of external macular support. In addition, a comprehensive long-term review by Alkabes and Mateo [16] highlighted the durability of macular buckling in myopic traction maculopathy by demonstrating sustained anatomical success through modification of posterior scleral contour. Further support for this biomechanical concept comes from the work of Mura et al. [17] who reported favorable anatomical and functional outcomes using a T-shaped macular buckle, with or without vitrectomy, in highly myopic eyes with complex macular pathology, including schisis and retinal detachment. Although BLD in nAMD and DME differ pathogenetically from myopic traction maculopathy, the biomechanical principle of external macular support may be relevant in chronic cases where persistent fluid and structural instability limit spontaneous resolution despite repeated anti-VEGF therapy.

Unlike previously reported techniques targeting BLD solely through subretinal fluid aspiration [13, 14], our combined ab interno and ab externo approach may offer a more comprehensive surgical strategy for selected cases of chronic, treatment-refractory BLD. Careful preoperative OCT assessment for patient selection is essential. Based on our experience, this combined technique appears most suitable and effective for eyes in which intraretinal fluid is organized into one or a few large, well-demarcated cystoid cavities clearly identifiable on preoperative OCT. In these cases, discrete fluid pockets allow controlled cannula entry, precise evacuation under iOCT guidance, and accurate macular buckle positioning. This observation is consistent with prior surgical reports showing that direct cyst fluid aspiration guided by iOCT is more feasible and reproducible in the presence of large, clearly delineated cystic spaces, implying that well-defined fluid pockets are technically more amenable to intervention compared to diffuse or poorly demarcated fluid collections [14]. Conversely, diffuse, multilobulated or poorly defined intraretinal fluid may limit surgical precision, present technical challenges and reduce predictability of outcomes. In all three cases, immediate and complete anatomical resolution of the BLD was achieved without intraoperative or postoperative complications. However, visual acuity gains at six month were modest, likely reflecting advanced and possibly irreversible outer retinal layers and RPE damage highlighted on preoperative OCT. Interestingly, although formal visual field testing or microperimetry was not performed, all patients reported notable subjective improvement in central visual disturbances and reduction of scotoma in daily activities, suggesting that even partial restoration of macular architecture may yield meaningful functional benefits. The absence of objective functional testing represents an important limitation, and subjective visual improvements should be interpreted cautiously. Nevertheless, this dissociation between anatomical and functional outcomes reinforces the concept that chronic BLD may compromise photoreceptor integrity and visual potential [4]. These findings suggest that the timing of intervention may be a critical determinant of outcome. In refractory bacillary layer detachments characterized by large, well-demarcated cystoid cavities, prolonged persistence of bacillary separation may contribute to progressive outer retinal degeneration. At the same time, our observations reinforce that anatomical resolution of BLD does not necessarily translate into functional recovery, as photoreceptor and retinal pigment epithelium damage may already be irreversible at the time of intervention. While our data do not allow conclusions regarding long-term functional benefit, they raise the hypothesis that earlier surgical collapse of large BLDs, prior to irreversible photoreceptor loss, may better preserve macular architecture in carefully selected patients; however, this remains speculative and requires further investigation. The risk–benefit profile of this combined approach must therefore be carefully considered. Given the limited functional improvement observed and the advanced disease stage in our cohort, surgical intervention should be reserved for highly selected, symptomatic cases not responsive to conventional therapy, and should be regarded as a salvage strategy rather than a standard treatment option. This study should be interpreted as a preliminary technical feasibility case series. Moreover, the relatively short follow-up period further limits assessment of long-term anatomical stability, recurrence, and functional outcomes.

## Conclusions

In this pilot case series, the combination of intraoperative OCT-guided subretinal drainage and macular buckling was found to be technically feasible and resulted in immediate anatomical resolution of large, refractory bacillary layer detachments. However, functional improvement was limited, likely reflecting irreversible outer retinal damage in advanced stages of disease. Accordingly, this approach should be considered only in highly selected symptomatic patients as a salvage intervention. Given the small sample size and short duration of follow-up, no conclusions can be drawn regarding long-term anatomical stability, recurrence, or functional outcomes. While these preliminary findings suggest that earlier intervention in carefully selected cases may help preserve outer retinal architecture, this hypothesis remains speculative. Further studies are required to better define the indications, optimal timing, and overall clinical value of this combined surgical strategy in refractory exudative macular disease.

## Supplementary Information

Below is the link to the electronic supplementary material.

Supplementary Material 1

## Data Availability

The datasets used and/or analyzed during the current study are available from the corresponding author upon reasonable request.
